# Social Media and Dermatology During the COVID-19 Pandemic: Analyzing User-Submitted Posts Seeking Dermatologic Advice on Reddit

**DOI:** 10.7759/cureus.33720

**Published:** 2023-01-12

**Authors:** Nader Aboul-Fettouh, Kevin P Lee, Natalie Kash, Kathleen Kroger, Sirunya Silapunt

**Affiliations:** 1 Dermatology, Mayo Clinic, Rochester, USA; 2 Dermatology, University of Texas McGovern Medical School at Houston, Houston, USA; 3 Dermatology, Root Hair Institute, Bellevue, USA; 4 Dermatology, University of Texas Medical Branch at Galveston, Galveston, USA

**Keywords:** skincareaddiction dermatologyquestions, advice, social media, online, dermatology, reddit

## Abstract

Introduction: Reddit, a popular social media website, has numerous forums where users may discuss healthcare-related topics and request diagnostic and treatment advice for dermatologic conditions. We sought to analyze and grade user-submitted requests for dermatologic advice and their top responses on Reddit.

Methods: User-submitted posts requesting diagnostic advice and their respective responses on two popular Reddit forums, SkinCareAddiction (ScA) and DermatologyQuestions (DQ), were reviewed by three board-certified dermatologists using a grading rubric designed for this study.

Results: 300 posts and comments were reviewed. Diagnoses among all graders matched in 52.3% of posts with a mean grader confidence score of 4/5 (95% CI 3.89-4.11). 31% of responder’s comments recommended a diagnosis not included by any reviewer. Mean scores for the top comment’s accuracy, appropriateness, and potential to be misleading/dangerous were 3.28/5 (95% CI 3.12-3.45), 3.3/5 (95% CI 3.14-3.45), and 2.33/5 (95% CI 2.18-2.48), respectively.

Conclusion: Reddit may be informative to patients requesting dermatologic advice. However, responses should be taken with caution as the information provided may be inaccurate or insufficient for treatment recommendations. Dermatologists should be aware of these resources used by patients.

## Introduction

Social media has become an increasingly popular source of news and information. In 2015, 90% of young adults in the United States with internet access used social media [1]. A survey by the Harris Poll in May 2020 showed that during the COVID-19 pandemic, 46% to 51% of US adults were increasing their social media usage [2]. Through popular social media sites such as Facebook, Twitter, TikTok, and Reddit, millions of users can discuss and comment on a wide variety of topics, including medicine and healthcare. This vast pool of information can be useful to patients seeking additional information and recommendations on their personal medical diagnoses. This avenue may have become more popular at a time when the US dermatology practices experienced a significant decrease in office visits due to COVID-19 [3]. However, there lies a significant challenge in validating medical advice posted online, where misinformation can be present. On most of these websites, any person can create an account and freely post advice and recommendations. 

On the social media site Reddit, content is user submitted and may include text-based posts, links, images, or videos. A ranking algorithm determines the visibility of each post and its associated comments based on 'upvotes' and 'downvotes' - actions a user takes to like a specific post or comment and increase its visibility to other Reddit users. In 2020, Reddit had accumulated 42 million daily active users, 303.4 million posts, two billion comments, and 49.2 billion upvotes [4]. Reddit is separated into topic-specific subreddits, which range from discussions on fitness (‘r/fitness’), science (‘r/science’), and even niche topics such as wedding planning (r/weddingplanning). Unsurprisingly, numerous health-related subreddits have become quite popular. Two subreddits, Dermatology Questions (DQ)(r/dermatologyquestions) and SkinCareAddiction (ScA)(r/skincareaddiction), have 22.1 thousand and 1.5 million users, respectively [5,6]. These subreddits are home to a variety of posts about skin conditions and treatments. In a 2018 study, Lawrence et al. showed that 33% of posts on ScA mentioned seeking medical advice [7]. The DQ subreddit is specific to users requesting diagnostic help about their skin concerns from a dermatologist or medical professional, who may have a ‘verified’ tag if approved by the subreddit’s moderators. The list of moderators on DQ and ScA is public, although only their Reddit alias is shared, which excludes any personal information about the user. However, any user is free to comment and respond to any post. Many posts include a photograph along with the post. Rules on these subreddits request users to report “all bad or potentially harmful advice,” although “well-intended but incorrect advice should not be reported."

In this study, we analyzed and graded user-submitted photos requesting diagnostic help on DQ and ScA and their top responses in order to better characterize the accuracy and reliability of dermatologic advice received on Reddit.

## Materials and methods

Data collection

User-submitted posts to ScA and DQ with a photograph and a request for diagnostic help were included in this study. Posts without any comments that provided a diagnosis were excluded. The website, Pushshift Reddit Search (https://redditsearch.io), was used to identify posts from ScA, using “skincareaddiction” for the subreddit and “skin concerns” as the search term [8]. For DQ, posts were identified directly from the “Hot” section of the forum, which is updated regularly through the Reddit post visibility algorithms. Posts on DQ and SCA were retrospectively reviewed until 150 eligible posts from each subreddit were collected, for a total of 300 posts. The photographs, the history provided by the original poster, and the highest ‘upvoted’ comment that included a diagnosis were extracted from eligible posts.

Post-analysis

**Figure 1:**
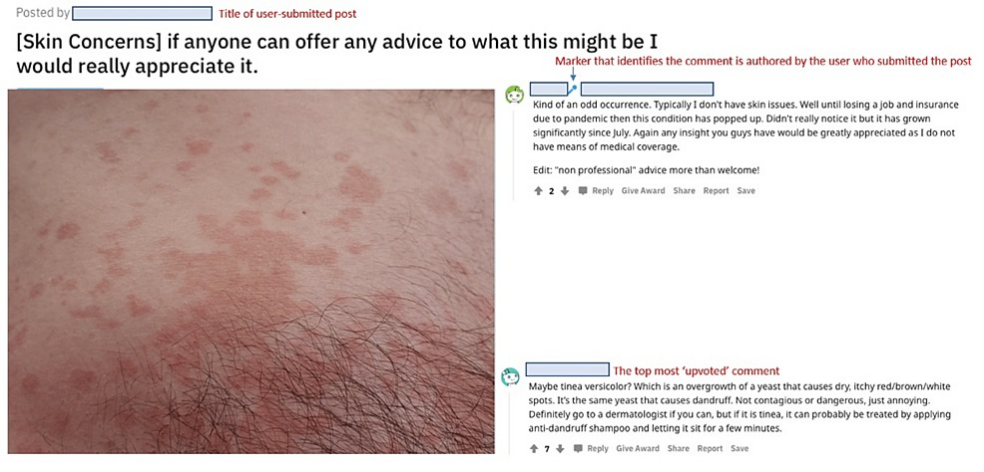
Example of a user-submitted post requesting a dermatologic diagnosis

PowerPoint slides were created for the posts collected. Each slide presented the user-submitted photo from the Reddit post, any information provided by the author to help in diagnosis, as well as the top comment that provided a diagnosis (Figure 1). Three board-certified dermatologists (authors KK, NK, SS) reviewed each post by answering a set of questions for each slide (Table 1). The data points included: the rating of image sharpness and color (1 poor to 4 excellent); diagnosis; confidence score for diagnosis (1 unsure to 5 confident); recommendation of a PCP visit, dermatology visit, or neither; recommendation of prescription medication, over-the-counter medication, or neither; warrant a biopsy (yes or no); whether the lesion or rash in the photo seemed acutely concerning (defined as a recommendation for the patient to be seen by a physician within two weeks, yes or no); and grading of the top Reddit comment for accuracy (1 low to 5 high), appropriateness (1 low to 5 high), and whether it may be misleading or potentially dangerous (1 low to 5 high).

**Table 1:** Grading scale for analysis of Reddit posts

Criteria	Grade				
Subjective ratings of image sharpness and color	1 poor	2 fair	3 good	4 excellent	
Best guess diagnosis					
Confidence score from 1 (unsure) to 5 (confident)	1	2	3	4	5
Recommend a PCP visit or Dermatology visit?	PCP	Derm	None		
Warrant prescription medication?	Prescription	OTC	None		
Warrant a biopsy? (Yes/No)	Yes	No			
Acutely concerning? (recommended to be seen in 2 weeks)	Yes	No			
Reddit comment (accurate) (1=low, 5=high)	1 low	2	3	4	5 high
Reddit comment (appropriate) (1=low, 5=high)	1 low	2	3	4	5 high
Reddit comment (misleading/potentially dangerous) (1=low, 5=high)	1 low	2	3	4	5 high

## Results

A total of 300 posts (150 from DQ and 150 from ScA) and their respective top comments were collected. For DQ, the timestamps of the posts collected spanned from April 10, 2021, to April 29, 2021. For ScA, the timestamps spanned from October 16, 2020, to May 1, 2021. Pooled results of the post-analysis by the three board-certified dermatologists are summarized in Table 2. The three graders provided the same diagnosis for 52.3% of the posts (157/300), with an average grader confidence score of 4 (95% CI 3.89-4.11) on a 5-point scale. For 31% (93/300) of the posts, the top responder’s comments recommended a diagnosis not provided by any of the three graders from our study. The average subjective rating for the image sharpness and color of the photos was 2.82 (95% CI: 2.73-2.91) on a 4-point scale, with 4 being the highest score achievable. The graders recommended a dermatology visit for 88.6% of the posts. The graders recommended a prescription medication for 65.7% of the posts, an over-the-counter medication for 8.8% of the posts, and neither prescription nor over-the-counter medication for 25.6% of the posts. A biopsy was recommended for 9.4% of the posts, and 5.2% of the posts were deemed acutely concerning by the graders. The top Reddit comments were rated for accuracy and appropriateness on a 5-point scale, where a score of 5 correlated to the highest level of accuracy and appropriateness in response to the clinical data. The mean accuracy and appropriateness of the posts were 3.28 (95%CI 3.12-3.45) and 3.3 (95% CI 3.14-3.45), respectively. On a 5-point scale where 5 correlated with a comment that was highly misleading/potentially dangerous, the average Reddit comment grade was 2.33 (95% CI 2.18-2.48).

**Table 2:** Redditors vs. Dermatologists: Analysis of user-submitted posts and their respective top ‘diagnostic’ comments to the ScA and DQ subreddits Note that 150 posts from r/DermatologyQuestions and 150 posts from r/SkinCareAddiction were reviewed for a total of 300 posts. For analysis, 3 graders reviewing 300 posts amounted to a total of 900 data points. ScA = r/SkinCareAddiction subreddit; DQ = r/DermatologyQuestions subreddit; PCP = primary care physician; OTC = over-the-counter

	Combined (n or 95% CI)	ScA (n or 95% CI)	DQ (n or 95% CI)
Best guess diagnosis: All 3 graders agreed on a diagnosis	52.3% (157/300)	56.7% (85/150)	48.0% (72/150)
Subjective ratings of image sharpness and color	2.82 (2.73-2.91)	2.64 (2.49-2.78)	3.00 (2.89-3.12)
Confidence score from 1 (unsure) to 5 (confident)	4.00 (3.89-4.11)	3.95 (3.78-4.11)	4.05 (3.90-4.20)
Recommend a PCP visit or Dermatology visit?	Derm 88.6% (797/900)	Derm 86.0% (387/450)	Derm 91.1% (410/450)
	PCP 4.6% (41/900)	PCP 6.0% (27/450)	PCP 3.1% (14/450)
	Neither 6.9% (62/900)	Neither 8.0% (36/450)	Neither 5.7% (26/450)
Warrant prescription medication?	Prescription 65.7% (591/900)	Prescription 68.2% (307/450)	Prescription 63.1% (284/450)
	OTC 8.8% (79/900)	OTC 8.7% (39/450)	OTC 8.9% (40/450)
	Neither 25.6% (230/900)	Neither 23.1% (104/450)	Neither 28.0% (126/450)
Warrant a biopsy? (Yes/No)	Biopsy 9.4% (85/900)	Biopsy 4.4% (20/450)	Biopsy 14.4% (65/450)
	No Biopsy 90.6% (815/900)	No Biopsy 95.6% (430/450)	No Biopsy 85.6% (385/450)
Acutely concerning? (recommended to be seen in two weeks)	Acutely concerning 5.2% (47/900)	Acutely concerning 7.5% (34/450)	Acutely concerning 2.8% (13/450)
Reddit comment (accurate) *(1=low, 5=high)*	3.28 (3.12-3.45)	3.25 (3.01-3.48)	3.32 (3.09-3.55)
Reddit comment (appropriate) *(1=low, 5=high)*	3.30 (3.14-3.45)	3.24 (3.02-3.47)	3.35 (3.13-3.57)
Reddit comment (misleading/potentially dangerous) *(1=low, 5=high)*	2.33 (2.18-2.48)	2.20 (1.99-2.40)	2.33 (2.17-2.48)

## Discussion

In 2020, the number of social media users surpassed the 3.8 billion mark worldwide [9], and its use has increased since the COVID-19 pandemic began [2]. There has been an increased interest in the role of social media, especially in healthcare [10,11]. Several studies have explored healthcare-related topics, including gout, radiology, and arthritis, on Reddit [12-14]. These studies focused on categorizing the posts and their respective comments to analyze the type of discussions and questions occurring on the subreddits rather than reviewing the content for accuracy and reliability. In 2016, an analysis of 25 health discussion threads selected across three websites (Reddit, Mumsnet, and Patient) on the topics of HIV, diabetes, and chickenpox found most of the information posted online by qualified medical doctors and nonmedically qualified respondents to be of reasonable quality [15]. In this study, we aimed to characterize the quality of the diagnostic information and advice crowdsourced by online commenters on the social mediate site Reddit, as determined by board-certified dermatologists.

Of the 300 posts reviewed in this study, the board-certified dermatologist graders agreed on the same diagnosis 52.3% of the time, with a mean confidence score of 4/5. The discrepancy among the graders may be due to the suboptimal quality of the images provided by the patients and the limited history available online. The graders recommended 88.6% of the time that the original poster should have an in-person dermatology visit for further evaluation. If the information provided by the users was sufficient to make an accurate and reliable dermatologic diagnosis, we would expect our graders to have a higher concordance rate in their diagnoses. Users requesting advice on these forums should be aware that an accurate and reliable diagnosis is difficult to make, and any advice received should be interpreted with caution.

In some regards, these types of forums and posts qualify as telemedicine, specifically store-and-forward (SAF) teledermatology, where clinical photos and information are forwarded by a referring provider to a dermatologist for aid in triaging and diagnosing [16]. Although teledermatology is a useful tool when in-person examinations are not readily available, studies have shown limitations in its diagnostic accuracy. In 2001, Lim et al. found a concordance rate of 79% (range 73%-85%) among five dermatologists in their primary diagnoses when reviewing SAF teledermatology cases that excluded patients with warts and acne [17]. A similar study at the Gazi University Department of Dermatology found the rate of agreement among three teledermatologists to range from 44% to 70% [18]. When shifting the viewpoint to accuracy, teledermatology still has significant limitations. Gerhardt et al. assessed the diagnostic accuracy of teledermatology consultations at a local Veterans Affairs dermatologic clinic and found only a 75.3% concordance of teledermatology diagnosis. 60.2% of patients had additional diagnoses when examined in person [19]. In these studies on teledermatology, the photos and clinical information routed to the dermatologist were sent by the referring providers rather than the patients themselves. In our study, the three board-certified dermatologists only reached a 52.3% concordance rate in their diagnoses. This is not unexpected as the photos and clinical history provided by Reddit users would be expected to be of lower quality compared to those from referring providers. Thus, Reddit users should be cautious of diagnoses and treatment recommendations received on Reddit, especially when given by non-verified responders.

The comments in response to users’ posts on these subreddits were also graded. In 31% of the posts, the diagnosis in the top comment did not match any of the diagnoses made by our graders. However, our study showed that many of the comments were of reasonable quality in appropriateness and accuracy. Few comments were deemed to be significantly misleading or potentially dangerous. It is important to note that the comments did not all hold equal value. Some commenters chose to clarify their background before providing a diagnosis. Often, users started their posts with “NAD”, short for “Not a doctor” so others could take their advice with a sense of caution. On DQ, one specific user is a board-certified dermatologist (verified by the subreddit’s moderators), and this is noted on the subreddit's 'about' page. Comments from medical professionals would skew the data as more accurate and reliable responses would be expected.

Resources such as ScA and DQ can aid patients in obtaining information about various dermatologic concerns, but caution should be taken when seeking diagnoses and definitive answers. The majority of comments reviewed in this study scored higher in the appropriateness and accuracy categories compared to being misleading or potentially dangerous. Many patients have limited access to in-person office visits with dermatologists due to insurance, long wait times, geographic location, or other factors [20-22]. Understandably, subreddits such as ScA have accumulated over 1.3 million users that post an average of 881 comments per day. In addition to diagnoses, ScA offers discussion on a much broader range of topics, including skin care products, personal experiences within skincare and dermatology, and even sharing humorous posts and jokes on relevant topics. The goal of our study was not to discourage posts or discussions from occurring but rather to better inform patients and physicians about the quality and reliability of advice posted online. In our study, we found that an in-person visit with a board-certified dermatologist was often recommended. Some patients would need a biopsy to help with diagnosis, and many would require treatments with prescription medications. Dermatologists and healthcare providers should be aware that these discussions and posts are occurring, to better inquire about them when evaluating patients. Patients may not freely disclose that they sought and potentially followed medical advice they received online.

There are several limitations to our study. The first limitation is that there are numerous dermatology-related forums on Reddit and other social media platforms that were not included in our study [23]. Furthermore, the grading rubric we assembled has not been standardized. Grading whether a comment was ‘appropriate’ or ‘misleading’ was open to interpretation by the dermatologist reviewing the post. The reviewers were board-certified dermatologists reviewing cases to determine whether the help of a board-certified dermatologist in the clinic is needed, a potential conflict of interest. As the top comment was available to the graders when the user-submitted photo was reviewed, one could argue that the graders could have been biased to lean toward that diagnosis. However, there was little reason for the graders to trust the comment’s reliability. None of the graders were aware that one of the commenters was possibly a verified medical professional. As data analysis was not stratified by image quality, the correlation between image quality and diagnostic accuracy was not calculated. This could be expanded upon in further studies to determine if users with higher-quality images allow for crowdsourced diagnoses to have higher diagnostic accuracy. Also, this study only used three board-certified dermatologist reviewers. Studies with a larger group of reviews could better quantify and characterize the online information. The 300 posts included in this study are a minuscule sample of the larger number of healthcare and medically-related discussions occurring online each day. Furthermore, the grading rubric used in this study has not been standardized.

## Conclusions

Resources such as ScA and DQ can aid patients in obtaining information about various dermatologic concerns, but caution should be taken when seeking diagnoses and definitive answers. Many patients have limited access to in-person office visits with dermatologists due to insurance, long wait times, geographic location, or other factors, especially during the COVID-19 pandemic. Dermatologists and healthcare providers should be aware that these discussions and posts are occurring to better inquire about them when evaluating patients that may not freely disclose that they sought and potentially followed medical advice they received online. While these resources can provide useful advice, patients must recognize there is potentially harmful and dangerous advice readily available online. We recommend that patients should strictly follow advice and care from healthcare providers or trusted online resources.
